# Bilateral total iris atrophy, corneal decompensation and glaucoma following bilateral cosmetic artificial iris implantation: A case report of severe sequela and successful management

**DOI:** 10.1097/MD.0000000000037457

**Published:** 2024-03-22

**Authors:** Wisam Shihadeh, Abdelwahab Aleshawi, Yara Aburamadan, Mohammed Al-Shalakhti

**Affiliations:** a Division of Ophthalmology, Department of Special Surgery, Faculty of Medicine Jordan University of Science and Technology, Irbid, Jordan; b University College London, Hospital NHS Trust, London, UK; c Faculty of Medicine, Yarmouk University, Irbid, Jordan.

**Keywords:** artificial iris implant, Boston keratoprosthesis, corneal edema, glaucoma drainage device

## Abstract

**Purpose:**

Cosmetic iris implants have a record of high ocular complications and are no longer in use. These complications include glaucoma, corneal decompensation, iris atrophy, uveitis, cataract and retinal detachment.

**Case presentation:**

We report a case of a 44-year-old lady presented with bilateral total iris atrophy, glaucoma and corneal decompensation after cosmetic artificial iris implantation. The patient underwent bilateral artificial iris removal, glaucoma drainage device for the right eye, and micropulse laser for the left eye. In addition, she underwent phacoemulsification with iris-diaphragm intraocular lens implant for the right. The cornea of the right eye ended up with successful Boston keratoprosthesis after rejection of previous 2 grafts.

**Conclusions:**

To the best of our knowledge, we describe the first report of bilateral total iris atrophy following a cosmetic iris implant accompanied by bilateral glaucoma and corneal decompensation.

## 1. Introduction

Silicone anterior chamber iris implants were first developed in 2006 and were used originally for the treatment of oculocutaneous albinism, as well as patients with iris defects such as hereditary iris coloboma or traumatic iris damage. The off-label use of these prosthetic iris devices for cosmetically changing eye color demonstrated serious complications with notoriously high rates of uveitis, glaucoma, and corneal decompensation.^[1,2]^ Two of the well-known iris prosthesis brands used in eye color change are NewIrisColor and the newer, supposedly modified for a lower rate of complications, BrightOcular. NewIrisColor design and complications have been described thoroughly elsewhere.^[1–3]^ BrightOcular iris prosthesis has patented posterior grooves that allow better flow of aqueous and minimal iris chaffing. This design theoretically decreases the risk of complications. However, Mansour et al reported the largest case series of 12 patients having had bilateral BrightOcular implants with a high rate of vision-threatening complications.^[4]^

In this article, we describe a case presented with major complications secondary to artificial iris implants 3 years following the surgery. To our knowledge, this is the first case report of artificial iris implant-induced total iris atrophy along with glaucoma and corneal decompensation.

## 2. Case presentation

A 44-year-old woman; not known to have any medical illness presented with a long complicated ophthalmic history. The past history of the patient refers back to 12 years ago. At that time, the patient underwent bilateral cosmetic artificial iris (BrightOcular) implantation in another country. Three years later, the patient started to develop bilateral progressive vision loss in both eyes (more in the right eye) accompanied with redness, pain, tearing and photophobia. At that time, the best corrected visual acuity was 6/40 in the right eye and 6/6 in the left eye. The intraocular pressure (IOP) was 60 mm Hg in the right eye and 38 mm Hg in the left eye on maximum medical therapy. Right eye exam showed moderate ciliary injection along with moderate diffuse stromal edema. Left eye exam showed moderate ciliary injection with mild diffuse stromal corneal edema. The light-blue iris prostheses were in situ in both eyes without visible iris tissue except for some black-colored peripheral anterior synechiae (PAS) more in the right eye. The lens showed cataractous changes more in the right eye. Gonioscopy revealed no visible angle structures 360° and the prosthesis plate was tucked in the angle recess with high and wide diffuse PAS scattered over the angles in both eyes. The optic nerve head exam showed a cup-to-disc ratio of 0.8 in the right eye and 0.5 in the left eye. The retinal exam was within normal limits.

**Figure 1.**
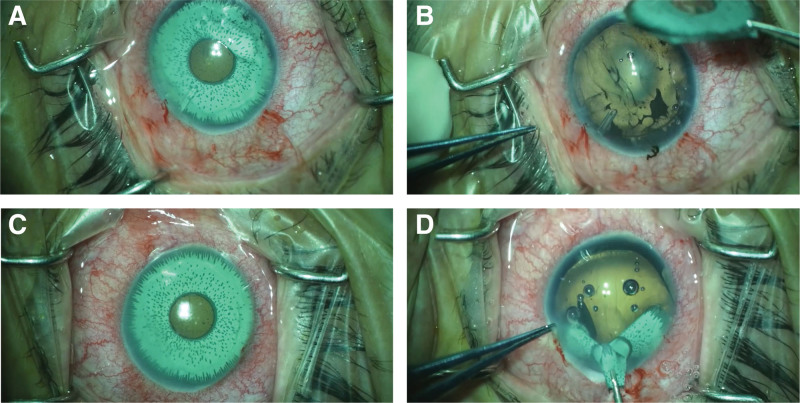
(A) This is the right eye with the cosmetic iris prosthesis, note the superior temporal tip of glaucoma drainage device. (B) The right eye after removal of the cosmetic iris prosthesis with total iris atrophy. (C) The same image for the left eye with the cosmetic iris prosthesis. (D) The left eye after removal of the cosmetic iris prosthesis with total iris atrophy.

A glaucoma drainage device (Ahmed Glaucoma Valve) for the right eye and micropulse diode laser transscleral cyclophotocoagulation were performed successfully. The IOP, thereafter, was maintained in low to mid-teens. One month later, she underwent bilateral iris prosthesis removal and to our surprise total iris atrophy was found in both eyes (Fig. 1). Three months later, phacoemulsification with implantation of iris-diaphragm intraocular lens was performed in her right eye. The right cornea continued to deteriorate despite adequate IOP control and hypertonic saline eye drops. Accordingly, the patient underwent keratoplasty twice (Descemet stripping automated endothelial keratoplasty and penetrating keratoplasty) for the right eye and both ended up with rejection and eventually failed opaque graft. Subsequently, a decision to proceed with Boston keratoprosthesis (type 1) implantation was made. Pros and cons as well as all concerns and potential complications were addressed.

Evaluation before the surgery revealed a visual acuity of counting fingers at 1 m distance in the right eye and 6/12 in the left eye. IOP was 9 mm Hg in the right eye and 12 mm Hg in the left eye. The right eye exam revealed a decompensated opaque graft and the iris-diaphragm intraocular lens in place. The left eye exam showed complete aniridia with cataractous lens. The anterior vitreous face was clear in both eyes. The posterior segment examination of the right eye was not possible as a result of the opaque graft, however; the B-scan was unremarkable. The posterior segment exam of the left eye revealed unremarkable retina and a cup-to-disc ration of 0.5.

**Figure 2.**
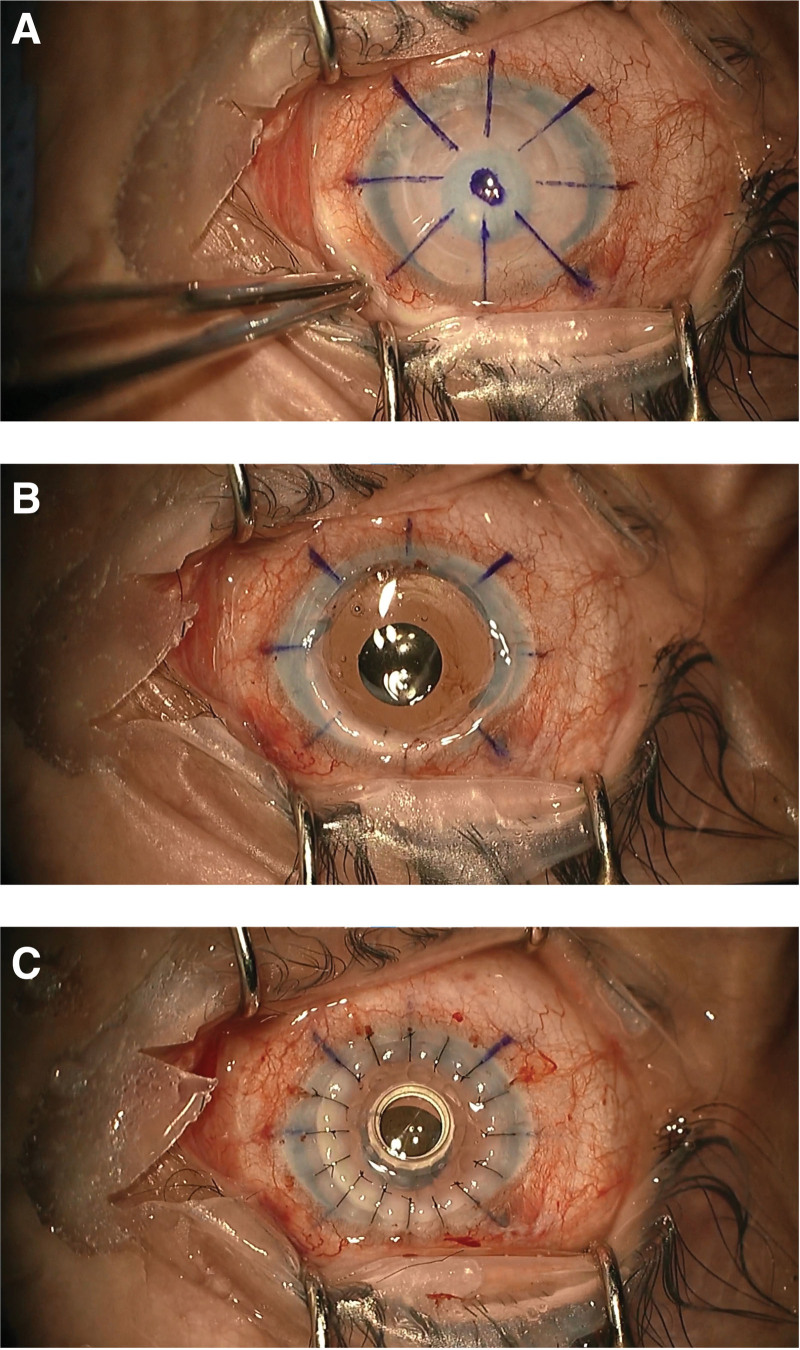
(A) The right rejected corneal graft before Boston keratoprosthesis implantation. (B) After removal of the rejected graft, note the iris-diaphragm IOL. (C) After implantation of Boston keratoprosthesis. IOL = intraocular lens.

Boston Type 1 keratoprosthesis for the right eye was performed under general anesthesia. The keratoprosthesis complex was sutured in place with 16 interrupted 10-0 nylon sutures (Fig. 2) (Video 1, http://links.lww.com/MD/L948). The surgery and postoperative course were uneventful.

On the very last follow-up visit (5 months after the surgery), the patient was doing very well with an uncorrected vision of 6/9, IOP was digitally fine and the keratoprosthesis was secured in place.

## 3. Discussion

To the best of our knowledge, this is the first case to report severe corneal decompensation, glaucoma and total iris atrophy following cosmetic artificial iris implantation (BrightOcular). Prosthetic iris devices in general are commonly used for the correction of congenital or traumatic iris defects. This justified medical use is not without complications, especially in phakic eyes. These complications include capsular rupture, transient or chronic inflammation, hypotony and retinal detachment, implant fracture, macular edema, glaucoma, endophthalmitis, iris damage and choroidal detachment, and corneal damage.^[5–10]^ Complications of these cosmetic iris implants could be much more common.

Hoguet et al reported that 7 out of 14 eyes which had NewColorIris implants had a raised IOP at presentation.^[11]^ The majority could not be managed with topical treatment alone and required surgical intervention with either trabeculectomy or aqueous drainage devices. Mansour et al reported 7 out of 12 patients in their BrightOcular series with elevated IOP.^[4]^ Mechanisms regarding the development of glaucoma include direct implant contact with both the trabecular meshwork and the iris resulting in continuous aqueous outflow mechanical damage and secondary pigment dispersion, respectively.^[12,13]^ The implant can also cause pupillary block if no prophylactic peripheral iridotomy is performed at the time of implantation. Other mechanisms include chronic anterior segment inflammation, chronic angle closure with PAS, and steroid-induced glaucoma. Our patient actually suffered from elevated IOP and advanced glaucoma bilaterally (more in the right eye) 3 years following the procedure. Procedures needed to control her glaucoma included glaucoma drainage device in the right eye and micropulse diode laser transscleral cyclophotocoagulation in the left eye.

Continuous chafing of the anterior surface of the natural iris with the rear surface of the iris prosthesis implant can result in secondary pigment dispersion and possible loss of iris stroma. This has been observed to different degrees with both NewColorIris and BrightOcular implants.^[1,12]^ Mansour et al reported patchy loss of anterior stromal pigment along the mid-periphery in 1 patient after explant, and sectoral iris atrophy with holes at the site of the implant hinges in another patient.^[4]^ However, it has never been previously reported that an artificial iris implant resulted in full-thickness loss of tissue in a complete circumferential manner as what we found in our patient. In a report by George et al, surgical aniridia during the explantation procedure was the cause of subtotal circumferential full-thickness iris tissue loss.^[14]^ We propose that the major arterial circle of the iris has been compromised possibly by mechanical compression from an oversized implant resulting in clean-cut coagulative necrotic “amputation” of her natural iris.

A review by Sikder et al found that 81.25% of phakic eyes developed corneal edema following artificial iris implantation.^[15]^ Houget et al reported 5/14 eyes with artificial iris implants developed corneal edema, only 1 of which resolved spontaneously following implant explantation.^[11]^ Mansour et al reported corneal edema and/or decompensation in 46% of the eyes, which were reversible in all cases after control of uveitis, lowering of IOP and explant surgery.^[4]^ Factors adding to corneal endothelial cell loss include glaucoma, iritis and implant-corneal touch at the periphery, especially in inappropriately sized implants. Our patient developed corneal decompensation in her right eye that did not improve even after the explantation of iris prosthesis and for which she needed 2 transplants that were rejected and ended up with Boston keratoprosthesis.

This case adds to the literature by highlighting the long-term sight-threatening sequelae of a new generation of artificial iris implants. We believe this is the first case report of complete circumferential iris atrophy secondary to artificial iris implantation. Unfortunately, the procedure lacks medical review or published evidence to support. We definitely counsel our patients against this procedure for the sole cosmetic alteration of iris color.

## Author contributions

**Conceptualization:** Wisam Shihadeh, Abdelwahab Aleshawi, Mohammed Al-Shalakhti.

**Data curation:** Wisam Shihadeh, Abdelwahab Aleshawi.

**Investigation:** Wisam Shihadeh, Yara Aburamadan.

**Methodology:** Wisam Shihadeh, Abdelwahab Aleshawi.

**Supervision:** Wisam Shihadeh.

**Validation:** Wisam Shihadeh, Abdelwahab Aleshawi, Yara Aburamadan, Mohammed Al-Shalakhti.

**Visualization:** Wisam Shihadeh.

**Writing – original draft:** Wisam Shihadeh, Abdelwahab Aleshawi, Mohammed Al-Shalakhti.

**Writing – review & editing:** Wisam Shihadeh, Abdelwahab Aleshawi, Yara Aburamadan.

## Supplementary Material


